# Influence of Niobium Content on the Chemical Composition, Microstructure, and Microhardness of Hardfacing Coatings Applied by SMAW

**DOI:** 10.3390/ma18245477

**Published:** 2025-12-05

**Authors:** Jaime Perez, Jesus Gutierrez, Jhon Olaya, Oscar Piamba, Americo Scotti

**Affiliations:** 1 Departamento de Ingeniería Mecatrónica, Facultad de Ingeniería y Ciencias Básicas, Fundación Universitaria Los Libertadores, Cra 16, N. 63A-68, Bogotá 111440, DC, Colombia; 2 Escuela de Diseño Industrial, Facultad de Artes, Universidad Nacional de Colombia, Cra 45, N. 26-85 Edificio Uriel Gutiérrez, Bogotá 111321, DC, Colombia; 3 Departamento de Ingeniería Mecánica, Facultad de Ingeniería y Ciencias Básicas, Fundación Universitaria Los Libertadores, Cra 16, N. 63A-68, Bogotá 111440, DC, Colombia; 4 Departamento de Ingeniería Mecánica, Facultad de Ingeniería, Universidad Nacional de Colombia, Cra 45, N. 26-85 Edificio Uriel Gutiérrez, Bogotá 111321, DC, Colombiaoepiambat@unal.edu.co (O.P.); 5 Center for Research and Development of Welding Processes, Federal University of Uberlandia, Uberlândia 38400-901, MG, Brazil; ascotti@ufu.br

**Keywords:** niobium, hardfacing, SMAW, welding

## Abstract

This study investigates the chemical composition, microstructural evolution, and mechanical behavior of hardfacing coatings produced by Shielded Metal Arc Welding (SMAW) using electrodes with varying niobium (Nb) contents (0%, 2%, 4%, 6%, and 8%), deposited at a constant current of 120 A and employing two- and three-layer configurations. Optical Emission Spectroscopy (OES) revealed a significant reduction in niobium transfer efficiency, with the Nb content in the coatings reaching up to 3.5 wt%, approximately 50% lower than in the electrodes. Chromium (Cr) content also decreased with increasing Nb additions due to the higher thermochemical affinity of niobium for oxygen, which promotes the formation of Nb oxides during welding. X-ray diffraction (XRD) analyses confirmed the presence of complex carbides, primarily NbC and M_7_C_3_-type Cr carbides, embedded in eutectic austenitic matrices. The incorporation of niobium promoted grain refinement and the precipitation of primary NbC carbides, particularly in multilayer coatings where dilution effects were reduced. Scanning Electron Microscopy (SEM) and Energy-Dispersive Spectroscopy (EDS) provided additional evidence, revealing an increased density of NbC particles and a concomitant reduction in CrC particle size with higher Nb contents. Microhardness testing showed a slight increase in hardness with increasing niobium, attributed to the higher intrinsic hardness and finer size of NbC particles. Overall, these findings highlight the role of niobium as an effective grain refiner and hard-phase promoter in SMAW-applied coatings, providing a foundation for optimizing wear-resistant overlays for demanding industrial environments.

## 1. Introduction

The development and optimization of welded hardfacing coatings constitute a fundamental strategy for improving the wear resistance of components operating under extreme service conditions [1]. Over recent decades, wear-related failures have accounted for a substantial portion of maintenance costs in sectors such as mining, power generation, and metalworking, driving increased interest in surface engineering technologies. Among these, hardfacing processes have emerged as cost-effective approaches to extend the service life of mechanical components exposed to abrasion, impact, and corrosion by producing protective layers with high hardness and superior tribological performance [2,3].

Among the various deposition techniques, Shielded Metal Arc Welding (SMAW) has proven to be an effective and accessible method for applying alloy-enriched coatings. Its versatility, low equipment cost, and operational simplicity make it especially suitable for field repairs and industrial maintenance, compared to processes such as Plasma Transferred Arc (PTA), Tungsten Inert Gas (TIG), or laser cladding [4]. The scientific literature reports substantial efforts to optimize SMAW-applied hardfacing coatings through the development of consumables incorporating strong carbide-forming elements such as chromium, vanadium, and titanium. These alloying additions promote the precipitation of hard, thermodynamically stable carbides that enhance microstructural integrity and improve resistance to abrasive and impact wear. However, the influence of consumable chemistry on carbide morphology, spatial distribution, and overall coating performance remains only partially understood, underscoring the need for further investigation to advance high-performance welded overlays [5,6].

The incorporation of elements such as niobium (Nb) and chromium (Cr) enables the formation of hard phases, including complex carbides (NbC, M_7_C_3_, and M_23_C_6_), which significantly improve the mechanical and tribological performance of the deposited material [7]. Chromium, widely used as a primary alloying element in Fe-based hardfacing electrodes, promotes the formation of M_7_C_3_ carbides, contributing to high hardness and moderate toughness. More recently, niobium additions have gained attention due to their ability to refine microstructures, stabilize carbides at elevated temperatures, and limit the formation of coarse primary carbides, which may otherwise serve as crack initiation sites [8,9]. These effects have been associated with improvements in hardness and wear resistance, particularly under severe abrasive or impact conditions.

In this context, the present study examines the microstructural and mechanical response of SMAW-applied hardfacing coatings produced with electrodes containing controlled niobium additions (0%, 2%, 4%, 6%, and 8%). All weld deposits were produced at a constant current of 120 A under rigorously standardized conditions to ensure process consistency. Unlike previous studies, which often examine niobium additions qualitatively or within restricted compositional ranges, this work provides a systematic assessment of the influence of incremental niobium content on carbide formation, grain refinement, and the evolution of wear-resistant phases. The findings offer new insights into the mechanisms through which niobium enhances coating performance, with direct implications for components operating in abrasive, corrosive, and high-demand industrial environments

## 2. Materials and Methods

The base electrode selected for this study was Overlay 62^®^ (Stoody/ESAB, Florence, SC, USA) [3], a high-alloy chromium–carbon electrode commonly used in Shielded Metal Arc Welding (SMAW) applications. It consists of an ASTM A36 [4] structural steel core and is typically employed as the final layer in components exposed to severe abrasive wear. Owing to its high chromium (Cr) and carbon (C) content, this electrode promotes the formation of chromium carbide–rich hardfacing layers. For the purposes of this investigation, niobium (Nb) was incorporated into the electrode formulation at nominal levels of 0%, 2%, 4%, 6%, and 8%.

Table 1 shows the chemical composition of the base electrode, Overlay 62^®^. It is worth noting its high chromium and carbon content, which produces a high density of chromium carbides in the deposited coating.

ASTM A36 steel [4] was selected as the substrate material. Its chemical composition is presented in Table 2. This steel is widely used in machinery and equipment fabrication due to its availability, ease of processing, and mechanical reliability. An additional advantage relevant to this study is its absence of alloying elements other than iron (Fe), carbon (C), and manganese (Mn), thereby minimizing potential interactions that could influence the chemical composition or microstructural evolution of the hardfacing coatings produced with varying niobium contents.

To control the amount of niobium introduced into the coating during electrode fabrication, samples were collected from electrodes containing niobium levels ranging from 0% to 8%. The coating material was pulverized and analyzed using an X-ray fluorescence (XRF) system. The corresponding chemical compositions are presented in Table 3.

After obtaining the different electrodes, these were applied onto ASTM A-36 structural steel plates, with dimensions of six inches (152.4 mm) by ten inches (254 mm) and a thickness of one-quarter inch (6.35 mm). This material was selected because it is commonly used in industrial repair and maintenance, and due to its low content of alloying elements, which was necessary since the goal was to evaluate the highest possible dilution in the steel in order to observe an extreme case. For the proper application of the coatings, the following procedure was followed:

1. The substrate plates were first cleaned by mechanical grinding using a wire-brush wheel to remove surface oxides and contaminants. Subsequently, the plates were washed with detergent and water to eliminate residual grease, followed by drying with compressed air. Once cleaned, the plates were transferred to the welding laboratory, where the hardfacing coatings were deposited under the predefined welding parameters (current, travel speed, and electrode–workpiece distance). These parameters were verified prior to deposition to ensure proper equipment performance. A Miller^®^ Syncrowave 250X SMAW (Miller Electric Mfg. LLC, Appleton, WI, USA) welding machine was used for all weld trials.
2. Weld deposition was carried out with particular attention to avoiding excessive heating of the substrate. The temperature of each plate was monitored using a Fluke^®^ IR 62 MAX infrared thermometer (Fluke Corporation, Everett, WA, USA) to ensure that the interpass temperature remained below 200 °C before applying subsequent beads. According to the experimental design, each plate received coatings consisting of two or three layers, applied at a constant current of 120 A and using electrodes formulated with niobium contents ranging from 0% to 8%.
3. After completing the two- and three-layer coatings, the surfaces were brushed mechanically to remove slag residues. The chemical composition of the final deposited layer was determined using spark optical emission spectrometry, performing four burns per sample. Measurements were conducted with a Baird DV4 spectrometer (Baird Atomic, Inc., Bedford, MA, USA).
4. Microhardness testing was performed using a Leco^®^ M-400-G2 microhardness tester (LECO Corporation, St. Joseph, MI, USA). Five indentations were taken per sample on the topmost coating layer, and the average value was reported. A pyramidal indenter with a 500 g load and a 15 s dwell time was employed.
5. Microstructural characterization was conducted using an FEI^®^ Quanta 200 scanning electron microscope (SEM) (FEI Company, Hillsboro, OR, USA). Images were acquired at magnifications ranging from 100× to 4000× to evaluate phase morphology and the influence of niobium on the microstructure. Energy-dispersive X-ray spectroscopy (EDS) analyses were performed on representative regions to determine local chemical compositions and to corroborate the results obtained from X-ray diffraction.

For this stage, an Xpert Pro^®^ (PANalytical B.V., Almelo, The Netherlands) system was used. Diffractograms were obtained for each of the established conditions using the θ–2θ method, ranging from 10° to 120°, with monochromatic Cu Kα radiation (λ = 1.5409 Å), 45 kV, 40 mA, and a step size of 0.02°. This complemented the information obtained from optical microscopy and SEM, supported by literature references, to finally establish the microstructure obtained for this type of deposit.

## 3. Results

### 3.1. Optical Emission Spectroscopy (OES)

OES analyses were performed on the coatings obtained at 120 Amperes, with three (3) layers and niobium contents of 0%, 2%, 4%, 6%, and 8%. Table 2 shows the results obtained for the chemical composition of the final layer. A reduction of nearly 50% was observed with respect to the niobium content in the electrode, with measured niobium contents ranging from 0.01 to 3.53 wt.%. The chromium content in the electrode before application was approximately 35%, while after the coating was produced, values ranged from 22.39% for the coating with 0% Nb to 18.4% for the coating with 8% Nb.

Hardfacing processes based on fusion welding generally exhibit varying efficiencies between the alloying element content in the electrode and the content found in the resulting coatings [5]. Part of these alloying elements is consumed in the generation of slag (in the case of SMAW processes) as well as in the formation of the protective atmosphere. Several variables or parameters influence this behavior, including amperage, voltage, deposition speed, and deposition technique [6,7,8,9]. The content of alloying elements, together with metallography and X-ray diffraction, provides useful evidence for understanding the formation mechanisms of the different carbides in coatings of this type [10,11].

Niobium and chromium are elements with different chemical properties and affinities for oxygen [12,13]. Niobium has a higher affinity for oxygen than chromium. This means that niobium tends to react more readily with oxygen to form niobium oxides, while chromium has a lower tendency to form chromium oxides.

Similarly to previous analyses on welding process efficiency [13], Hiraoka et al. [12,14] showed that niobium oxides of the type Nb_2_O_5_ have a Gibbs free energy of −346,100 cal/mol at 1000 K, whereas authors such as Ziemniak et al. [13] reported Gibbs free energy values for chromium oxides of the type Cr_2_O_3_ on the order of −203,155 cal/mol at temperatures near 1000 K. This suggests that at high temperatures, niobium has a greater affinity for the oxygen present in the welding atmosphere, which may result in metals reacting with atmospheric oxygen during the welding process and forming oxides. These oxides may also be present in welding fumes, depending on the degree of oxidation during the process.

Samples were taken from coatings obtained with electrodes containing two percent (2%) and eight percent (8%) niobium, applied in two (2) and three (3) layers, and their chemical composition was analyzed. This was done in order to evaluate the influence of applying additional layers. The results are shown in Table 3, where it can be observed that the change in the number of deposited layers affects the amount of niobium present in the resulting coating [15]. In welding, it is common to observe an increase in the amount of alloying elements as a function of the number of deposited layers. In most cases, this is because in the first layer(s) the dilution is greater, since the base material in this case lacks the elements provided by the coating, such as chromium (Cr) and niobium (Nb). As more layers are deposited, the weight percentage of these elements increases [16].

### 3.2. X-Ray Diffraction

**Figure 1:**
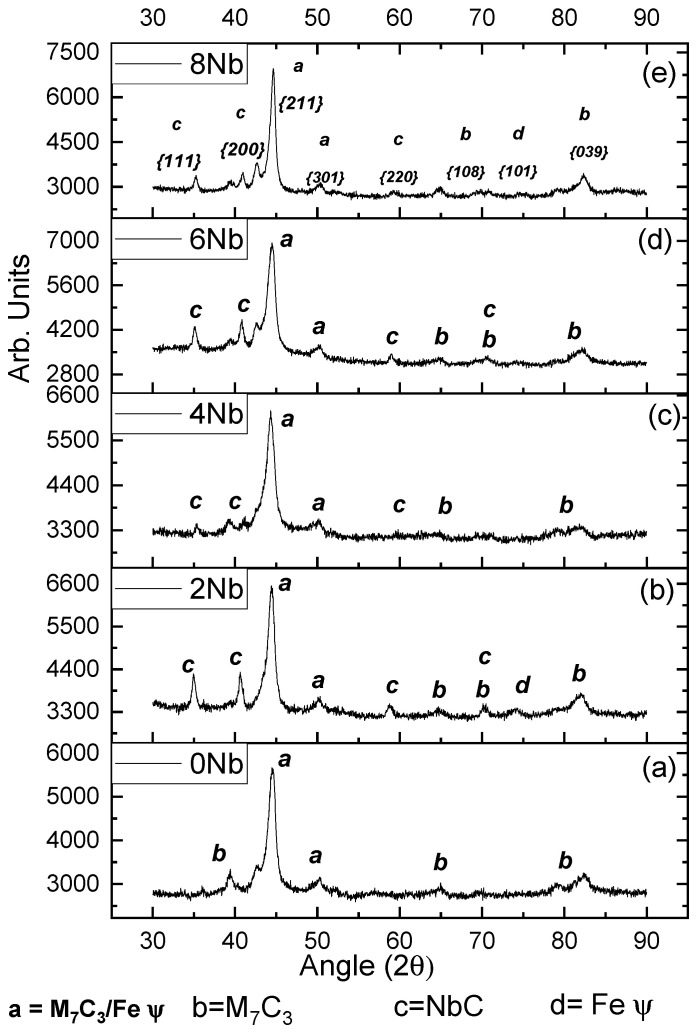
Diffractogram obtained from the coatings produced with the five (5) variations in niobium content. (**a**) 0% niobium content, (**b**) 2% niobium content, (**c**) 4% niobium content, (**d**) 6% niobium content, and (**e**) 8% niobium content.

From the samples obtained with the five niobium contents, using a current of 120 A and three (3) layers, X-ray diffraction (XRD) tests were carried out on the surface, resulting in the diffractograms shown in Figure 1. In this diagram, the diffractogram of the coating obtained with zero (0) percent niobium is shown along with the Miller indices. It was determined that it is mainly composed of M_7_C_3_-type carbides (chromium carbides and iron carbides) in a eutectic matrix, consisting of small M_7_C_3_-type carbides and austenite (Feγ). This agrees with the findings of other authors such as Cruz-Crespo et al. [17] and Chotěborský et al. [18]. The phase composition present in this coating is characteristic of deposits obtained with high chromium contents, above 15%, which provides the physical and mechanical properties sought in this type of material. Several authors have reported the existence of chromium carbides accompanied by various steel phases, including ferrite (Feα), austenite (Feγ), and cementite, generally located at the interface of chromium carbides [17,19,20,21,22,23,24,25,26,27]. According to Perez et al. [10], when analyzing coatings with three layers, no differences were observed in the peaks compared to the diffractograms obtained with a single layer.

In item 2 of Figure 1, the diffractogram was obtained for the coating generated with 120 amperes (120 A), three (3) layers, and two percent niobium content (2% Nb). The peaks from this coating revealed the additional formation, compared to item 1, of niobium carbides NbC {111}. This formation occurs due to the higher solidification temperature associated with this type of carbide [10], which allows these carbides to form and solidify at higher temperatures than chromium carbides. This is the basis for one of the main characteristics of niobium-added hardfacing coatings: refinement in carbide size, i.e., when niobium is added, the average size of carbides decreases [24,26,28,29,30]. Sha Liu et al. [31] established that the precipitation of niobium carbides occurs at higher temperatures than the formation of chromium carbides, approximately at 1350 °C, fifty (50) degrees above the temperature at which chromium carbides and the eutectic reaction begin to form. The eutectic reaction (liquid → eutectic M_7_C_3_ carbide + eutectic austenite) occurs at 1300 °C. Likewise, the initial formation of niobium carbides implies their presence within chromium carbides as grain refiners, making them a crucial factor in the refinement of grain size observed in coatings obtained with niobium additions.

In Figure 1, the diffractograms obtained from coatings generated with niobium contents of four percent (4%), six percent (6%), and eight percent (8%), respectively, at 120 amperes (120 A) and with three (3) layers, are shown. In these figures, the formation of both niobium (NbC) and chromium (CrC) carbides can be observed, with their respective Miller indices marked on the main peaks. Niobium carbides exhibit a preferential orientation of {111}.

### 3.3. SEM

**Figure 2:**
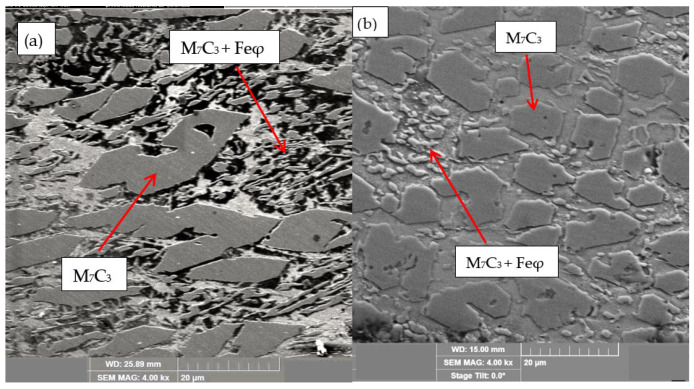
SEM of coating obtained with 0%Nb: (**a**) 120 A and 2 deposited layers, (**b**) 120 A and 3 deposited layers.

**Figure 3:**
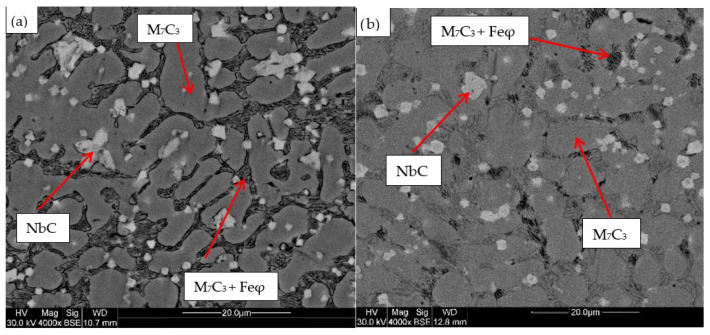
SEM micrograph of coating obtained with 2%Nb: (**a**) 120 A and 2 deposited layers, (**b**) 120 A and 3 deposited layers.

**Figure 4:**
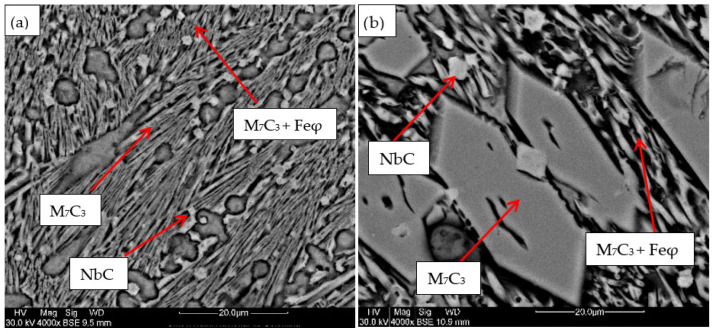
SEM micrograph of coating obtained with 4%Nb: (**a**) 120 A and 2 deposited layers, (**b**) 120 A and 3 deposited layers.

**Figure 5:**
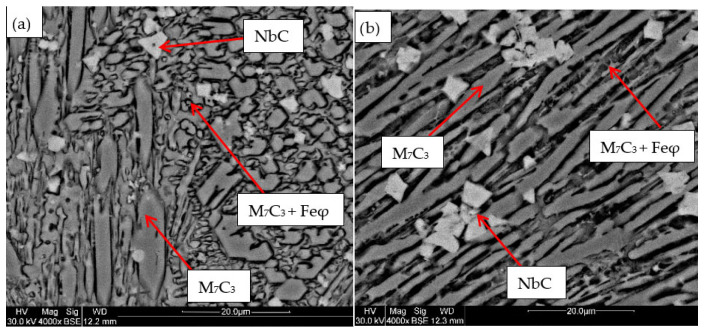
SEM micrograph of coating obtained with 6%Nb: (**a**) 120 A and 2 deposited layers, (**b**) 120 A and 3 deposited layers.

**Figure 6:**
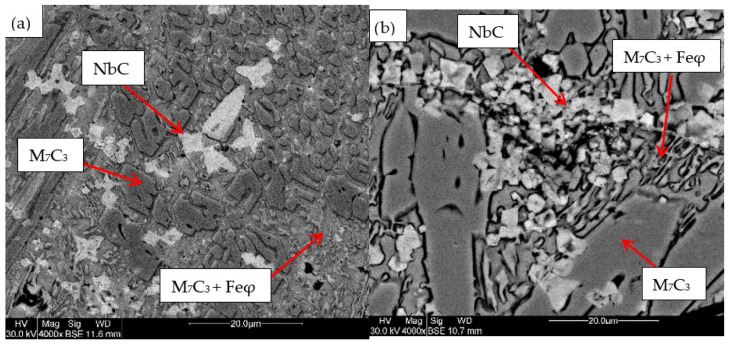
SEM micrograph of coating obtained with 8%Nb: (**a**) 120 A and 2 deposited layers, (**b**) 120 A and 3 deposited layers.

For the samples obtained from the experimental development, analyses were carried out using Scanning Electron Microscopy (SEM). Figure 2, Figure 3, Figure 4, Figure 5 and Figure 6 show the images obtained from the coatings applied at one hundred and twenty amperes (120 A), with two (2) and three (3) layers, for electrodes with zero (0%Nb), two (2%Nb), four (4%Nb), six (6%Nb), and eight percent niobium (8%Nb). It can be observed that with the increase of niobium in the electrode, niobium carbides (NbC) begin to appear, and as the niobium content increases, the size and quantity of these carbides also increase. Additionally, it is observed that the size of the chromium carbides decreases with increasing niobium content, corroborating the findings of authors such as Singla et al. [32], Correa et al. [25], Ripoll et al. [33], and Liu et al. [31], regarding the role of niobium as a grain size refiner.

According to the chemical composition found in Table 4 and Table 5, these coatings can be classified as hypereutectic alloys based on Fe-Cr-C ternary diagrams [34], which have been widely studied. For example, Bálsamo et al. [35] and Sadeghi [36] analyzed the interpretation of hardfacing deposits produced by welding using the liquidus surface in Fe-Cr-C ternary diagrams, where results similar to those presented here were obtained, predicting coatings rich in phases formed by primary M7C3 carbides in a eutectic-rich matrix consisting of γ-iron and small M7C3 carbides.

Based on the chemical composition obtained in Table 4, it is particularly interesting to observe the presence of titanium, especially in the niobium carbides, which agrees with the findings of Yang et al. [37], who determined a strong affinity of titanium for carbon compared to niobium. This suggests that titanium carbides act as nucleation sites for niobium carbides. This phenomenon is attributed to the higher solidification temperature of titanium carbides compared to niobium carbides.

Table 4 shows the chemical composition of the main microconstituents present in the hard coatings obtained with 120 amperes and three layers (wt%). It is important to note the presence of niobium in all M7C3-type carbides, demonstrating that this element plays a significant role as a primary former of this type of carbide, corroborating both theoretical and experimental findings reported by authors such as Liu et al. [31] and Cunha et al. [38]

### 3.4. Microhardness

**Figure 7:**
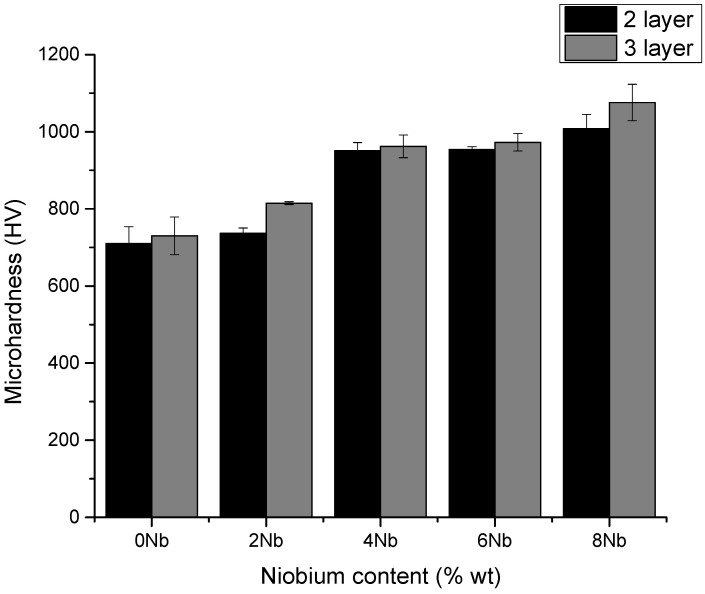
Microhardness test results for coatings obtained under conditions of 120 A, two (2) and three (3) layers, and variable niobium content.

Microhardness testing was carried out on the samples obtained with three (3) layers and a current of one hundred twenty amperes (120 A). The results are shown in Figure 7 and Table 6. It is worth noting that the values obtained showed a significant change, which is supported by the fact that, according to the analysis of the microstructure present, carbides were formed in the different chemical compositions—both chromium carbides (CrC) and niobium carbides (NbC). Chromium carbides, being larger in size, had a greater influence on the measured values. Niobium carbides, which are smaller in size, showed an increase in the measured microhardness values. This is consistent with the findings of Bedolla-Jacuinde et al. [39], Long Jeng et al. [28], Woydt et al. [40], among others, who reported that additions and increases in niobium content led to increases in measured hardness values, mainly due to the initial formation of niobium carbides and their higher hardness compared to chromium carbides.

## 4. Conclusions

The incorporation of niobium into Fe-Cr-C coatings resulted in a noticeable refinement of chromium carbide size. This effect can be attributed to the fact that niobium solidifies at higher temperatures than chromium compounds, thereby reducing the amount of carbon available for the subsequent growth of chromium carbides.

With respect to dilution, it was observed that niobium tends to decrease the dilution percentage. This behavior is associated with its role as a strong carbide-forming element, which contributes to limiting the melting of the substrate during the welding process.

The increase in niobium content promotes the formation of carbides that are harder than chromium carbides, resulting in higher microhardness values and confirming the positive effect of this element on strengthening the mechanical properties.

Across the tested range of niobium contents (0–8%), the coatings exhibited a progressive increase in microhardness. The baseline composition (0% Nb) showed the lowest hardness, governed primarily by chromium carbides. As niobium content increased from 2% to 8%, the formation of fine and hard niobium carbides intensified, leading to a steady and measurable rise in hardness. These results demonstrate that controlled Nb additions within this range effectively refine the microstructure and enhance the mechanical performance of the hardfacing coatings.

Note: The researcher will continue to address the comparative performance of electrodes with and without Nb, yet with two or three coating layers for abrasive and corrosion wear. This approach will enable a more in-depth analysis to determine the most suitable niobium content, if this is the case.

## Figures and Tables

**Table 1:** Chemical composition of the original deposited electrode (% by weight). Chemical composition of Overlay 62^®^ electrode [3].

C	Mn	Si	Cr	Ni	Mo	Fe
5.0	1.7	0.55	31	0.2	0.5	Balanced

**Table 2:** Chemical composition of ASTM A36 substrate (% by weight) [4].

Carbon	Manganese	Phosphorus	Sulfur	Silicon	Copper
0.27	0.6–0.9	0.04	0.05	0.4 máx.	0.2

**Table 3:** Chemical composition of the electrode coating with additions of 0%, 2%, 4%, 6%, and 8% niobium. Chemical composition of electrode coating (% by weight) [9].

	Fe	Cr	C	Nb	Si	Mn	Ti	Mo
Electrode 0%Nb	58.43	28.87	4.35	0	2.77	1.9	1.36	0.92
Electrode 2%Nb	61.22	26.38	3.84	1.83	2.43	1.64	1.20	0.92
Electrode 4%Nb	56.28	28.34	3.84	4.13	2.72	1.77	1.23	0.90
Electrode 6%Nb	57.73	26.80	3.84	5.65	2.72	1.53	0.98	0.84
Electrode 8%Nb	60.61	21.22	3.14	8.17	2.57	1.76	1.02	0.82

**Table 4:** Chemical composition obtained in the final deposited layer for the different niobium additions (% by weight).

Chemical Composition of Coating Obtained with Three Layers							
Electrode	Fe	C	Mn	Cr	Mo	Ti	Nb
0 Nb	67.08	5.34	1.36	22.39	0.96	0.84	0.01
2 Nb	67.23	4.33	1.39	21.85	0.95	0.81	1.04
4 Nb	63.96	5.12	1.24	23.47	1.04	0.78	2
6 Nb	64.9	4.43	1.26	22.52	0.95	0.75	2.62
8 Nb	68.56	3.58	1.34	18.4	0.98	0.74	3.53

**Table 5:** Comparative chemical composition between coatings with 2% and 8% niobium in two and three layers.

Chemical Composition Between Two and Three Layers					
Sample	Fe	C	Si	Cr	Nb
2%Nb 2 layers	88.55	3.79	2.67	2.51	0.979
2%Nb 3 layers	89.71	3.97	1.03	2.66	1.05
8%Nb 2 layers	85.48	3.66	2.61	2.04	4.75
8%Nb 3 layers	84.9	4.18	1.08	2.22	5.86

**Table 6:** Microhardness test results for coating obtained under conditions of 120 A, two, and three layers.

Microhardness Test Result for 120 A, 2 and 3 Layers		
Sample	HV	SD
0%Nb 2 layers	710.24	43.76
0%Nb 3 layers	730.51	48.76
2%Nb 2 layers	737.06	13.02
2%Nb 3 layers	814.89	3.81
4%Nb 2 layers	950.81	21.29
4%Nb 3 layers	962.21	29.57
6%Nb 3 layers	954.15	7.15
6%Nb 3 layers	972.58	22.68
8%Nb 2 layers	1008.07	36.91
8%Nb 3 layers	1075.96	47.21

## Data Availability

The original contributions presented in this study are included in the article. Further inquiries can be directed to the corresponding authors.
